# Macrophage plasticity in inflammatory and tumor microenvironments: mechanisms, evidence, and therapeutic opportunities

**DOI:** 10.3389/fimmu.2026.1949327

**Published:** 2026-10-22

**Authors:** Jun Zhang, Zhiwei Wang, Shuang Wu, Tao Meng, Ming Lu

**Affiliations:** 1 Division of Life Science and Medicine, Department of Colorectal Surgery, The First Affiliated Hospital of USTC (Anhui Provincial Tumor Hospital), University of Science and Technology of China, Hefei, Anhui, China; 2 Department of Immunology, School of Basic Medical Sciences, Anhui Medical University, Hefei, Anhui, China; 3 Department of Rheumatology and Immunology, The Second Affiliated Hospital of Anhui Medical University, Hefei, Anhui, China

**Keywords:** BACH1, inflammatory microenvironment, latent enhancer, macrophage plasticity, PU.1, tumor-associated macrophage

## Abstract

Macrophages are differentiated innate immune cells with niche-responsive plasticity and, in many resident populations, proliferative self-renewal. Single-cell omics, spatial profiling, lineage tracing, and epigenomic studies show that disease-associated macrophages can acquire collagen-producing, lipid-processing, vascular-construction, antigen-presentation, bone-resorptive, or fusion-associated functions. We use lineage-incongruent macrophage state as a descriptive umbrella for phenotypes that deploy a function or regulatory module conventionally associated with another cell type. Six phenomena are compared: functional adaptation, lineage-program borrowing, hybrid identity, intralineage differentiation, cell fusion, and candidate transdifferentiation. We distinguish state plasticity within the myeloid lineage, lineage-ambiguous or hybrid states with co-expression of different lineage programs, and transdifferentiation requiring established cellular origin, source-program attenuation, stable acquisition of a target-lineage network, and target-cell function. Evidence is graded from Level 1 marker observations, through Level 2 single-cell trajectory or spatial support and Level 3 lineage tracing, to Level 4 functional demonstration of the proposed converted identity. Under this framework, TREM2^+^ lipid-associated macrophages, osteoclasts, multinucleated giant cells, and most angiogenic macrophages represent specialization, intralineage differentiation, or fusion. TAM-to-CAF transitions are the strongest current candidates for cross-lineage conversion, but the evidence remains model- and organ-dependent, and causal human evidence is limited. A PU.1-anchored rewritable enhancer model provides a testable explanation for how macrophages retain a myeloid scaffold while deploying selected modules. Therapeutic proposals are therefore separated into mechanistic hypotheses, preclinical proof of concept, early clinical evidence, and established myeloid-niche therapies. State-selective targeting is promising, but clinical actionability requires validation of target stability, tissue-specific safety, biomarkers, and efficacy in adequately controlled human studies.

## 1 Introduction

The tissue-adaptive capacity of macrophages extends far beyond the conventional M1/M2 polarization framework. After entering an organ, macrophages undertake local tasks that include lipid clearance, iron recycling, extracellular-matrix processing, vascular maintenance, antigen presentation, and tissue repair. Their identity is therefore not sealed by a single immutable terminal program; instead, it combines a stable myeloid core with functional modules that remain responsive to the environment. Integrated tissue proteomic, transcriptomic, and epigenomic analyses show that organ niches preserve a shared macrophage framework while selectively engaging peroxisome proliferator-activated receptor-γ, liver X receptor, SMAD, MAF, and other tissue-dependent regulatory networks (1, 2). During chronic inflammation, metabolic stress, and cancer, this physiological adaptability can be amplified or co-opted, generating macrophage states that resemble fibroblasts, endothelial cells, B cells, or specialized lipid-processing cells. Many tissue-resident macrophages self-maintain through local proliferation (3). In allergen-induced type 2 inflammation, resident alveolar macrophages can lose PPARγ-dependent homeostatic programs and acquire inflammatory and fusion-associated states driven by interferon regulatory factor 4 (IRF4) (4).

These observations raise two related questions. First, does acquisition of a marker or function associated with another cell type constitute a breach of lineage identity? Second, do unusual macrophage states share an interpretable chromatin mechanism? Current evidence suggests that most examples do not involve wholesale replacement of myeloid identity. Rather, macrophages retain a regulatory scaffold maintained by PU.1/SPI1 and related factors while deploying selected gene modules also used by neighboring cell types. We therefore use lineage-incongruent state as a neutral descriptive umbrella. Here, we propose ‘lineage-incongruent macrophage state’ as a conceptual framework for this review, rather than as an established biological classification, to organize macrophage phenotypes displaying programs or features conventionally associated with other cell lineages. Physiological functional adaptation, osteoclast differentiation, and macrophage fusion are treated as comparator categories. We distinguish state plasticity (potentially reversible changes within the myeloid lineage), lineage-ambiguous or hybrid states (co-expression of programs from multiple lineages), and transdifferentiation (established origin and direction, progressive loss of the source program, stable acquisition of a target-lineage regulatory network, and target-cell function). Co-expression of M1- and M2-associated genes alone represents state plasticity, not lineage change.

We searched for peer-reviewed original studies published from 1 January 2018 through 22 August 2026. The start year was chosen to capture the period in which single-cell, spatial, and epigenomic approaches rapidly expanded the study of macrophage states, while retaining relevant papers from the full 2018 calendar year. The end date was the final search date. Core identity terms were ‘macrophage’ or ‘monocyte’. These were combined with phenotype terms (including lineage infidelity, transdifferentiation, myofibroblast, cancer-associated fibroblast, endothelial, vascular mimicry, B cell, osteoclast, and multinucleated giant cell), regulator terms (PU.1, BACH1, IRF8, BCL6, SMAD3, RUNX1, ASCL2, and PAX5), and method terms (chromatin, enhancer, single cell, spatial, and lineage tracing). The review focused on inflammatory-disease and tumor microenvironments. Studies were included if they were peer-reviewed original research, placed macrophages or monocyte-derived cells at a central mechanistic node, and contributed evidence on origin, regulation, spatial context, function, therapeutic perturbation, or an important counterexample. Evidence was weighted by experimental rigor. Particular weight was given to inducible or genetic lineage tracing, cell-type-restricted perturbation, orthogonal single-cell and spatial validation, multi-omic regulatory evidence, functional assays, replication across models, and confirmation in human tissue. Reviews, editorials, conference abstracts, preprints, M1/M2-only studies, and reports relying on a single marker or trajectory alone were not treated as evidence of fate conversion. The queries returned 760 unique records. After exclusion of non-original publication types and a mechanism-focused title/abstract screen, 107 high-relevance candidates remained. The database-derived synthesis included 75 original studies (**Figure 1**).

**Figure 1:**
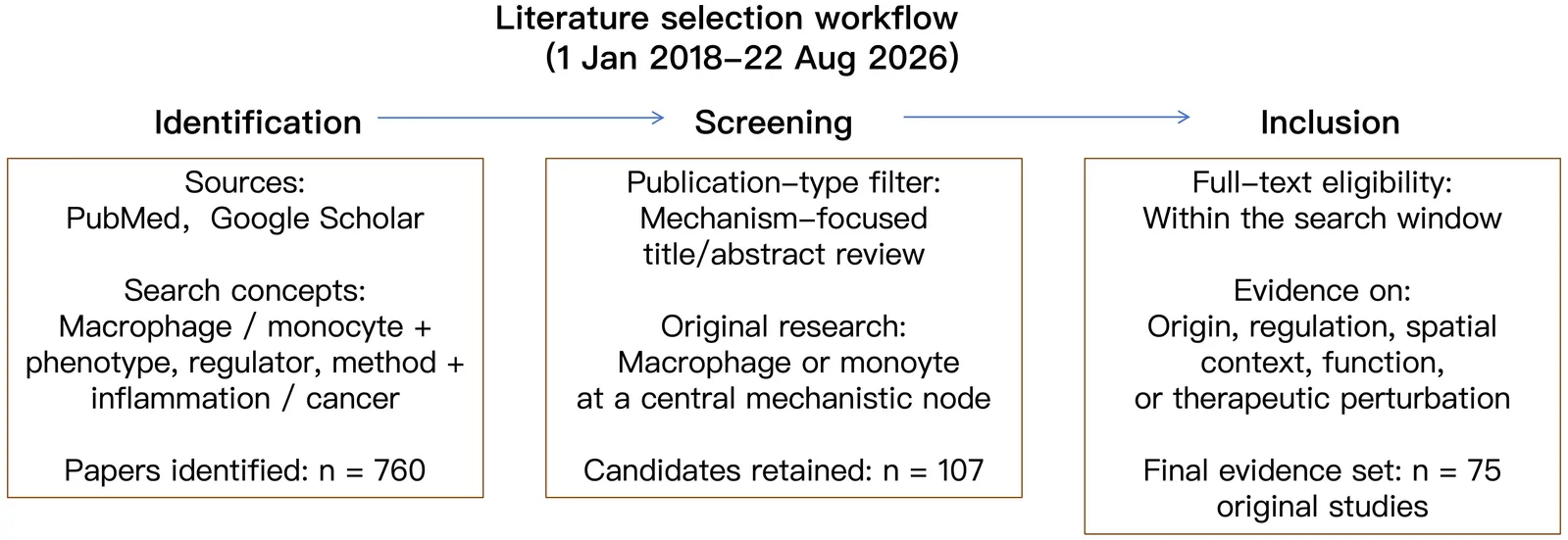
Literature selection workflow. PubMed and Google Scholar searches covering 1 January 2018 to 22 August 2026 identified 760 papers. Mechanism-focused screening retained 107 candidates, and full-text assessment yielded a final evidence set of 75 original studies.

## 2 Beyond polarization: macrophage plasticity and standards of evidence

### 2.1 Six biologically distinct phenomena and lineage interpretation

Expression of a non-myeloid marker by a macrophage does not by itself demonstrate a lineage transition. The lineage-incongruent state describes the observation to be explained; six mechanistically distinct phenomena must then be separated.

The first is physiological functional adaptation: the cell retains an unequivocal myeloid identity while intensifying a local function, as in TREM2-positive lipid processing, collagen deposition, or perivascular support (5–10). The second is lineage-program borrowing, in which a macrophage activates a transcription factor or surface molecule associated with another lineage without acquiring that lineage’s complete identity; PAX5-associated CD19^+^ TAMs are an example (11). The third is a hybrid-identity state, in which a myeloid core coexists with a coordinated cancer-associated fibroblast (CAF), vascular-construction, or mesenchymal module (12–18). The fourth is intralineage differentiation: monocyte–macrophage precursors differentiate into osteoclasts in response to RANKL (19). The fifth is cell fusion: mature macrophages form Langhans-type or foreign-body multinucleated giant cells (20). Neither osteoclastogenesis nor macrophage fusion crosses an embryonic lineage boundary. The sixth is bona fide transdifferentiation, which requires causal evidence for origin and direction, progressive attenuation of the source-lineage network, stable acquisition of a target-lineage regulatory network, and target-cell function. Functional adaptation represents state plasticity; program borrowing and hybrid identity require origin-resolved validation as lineage-ambiguous or hybrid states, whereas intralineage differentiation and fusion are comparator processes (**Figure 2**). Ruscitti et al. identified short-lived Ly6G-positive monocyte-derived macrophages during early recovery from influenza-induced lung injury that support alveolar epithelial regeneration, illustrating repair-associated specialization rather than lineage conversion (21).

**Figure 2:**
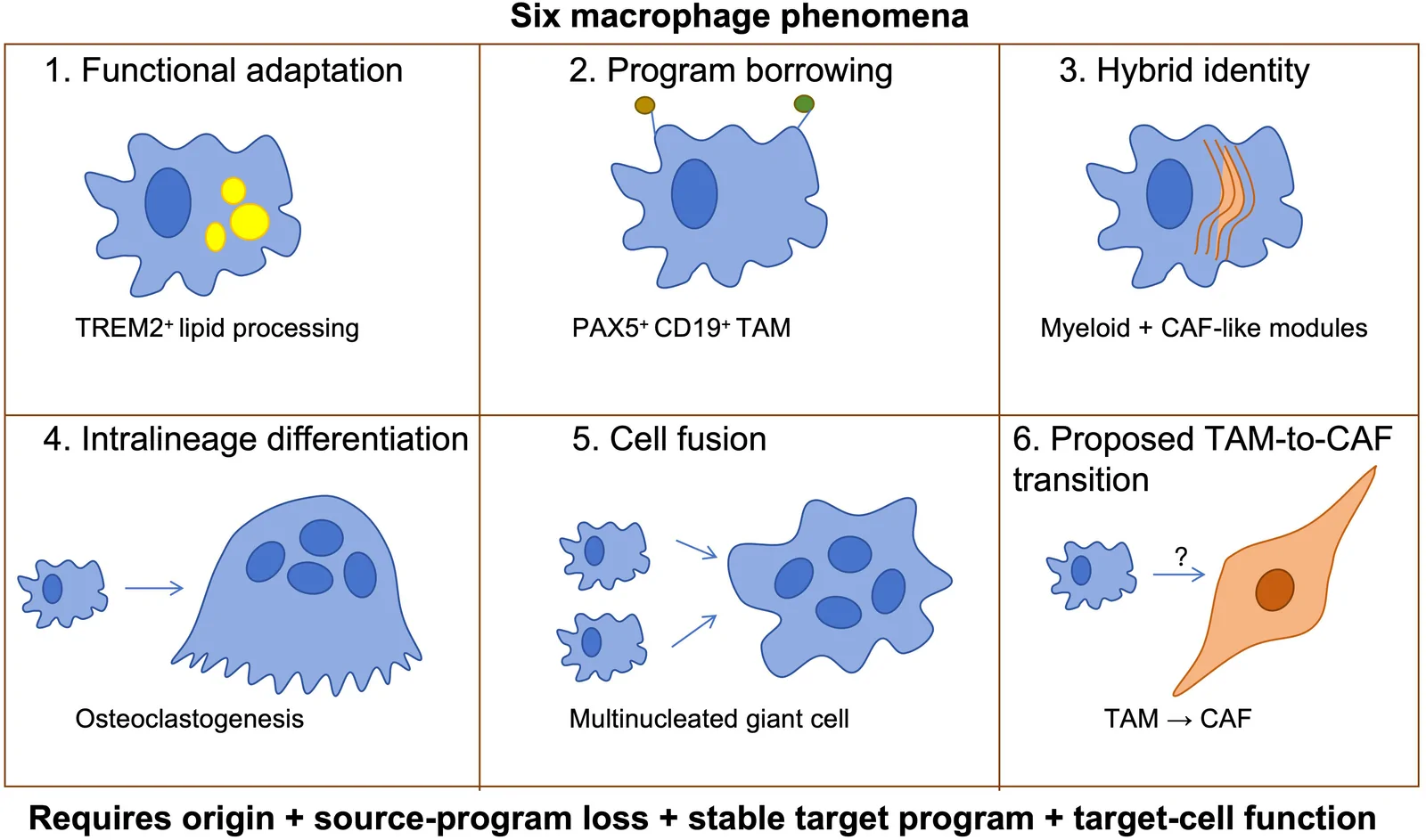
Six macrophage phenomena. The schematic distinguishes functional adaptation, program borrowing, hybrid identity, intralineage differentiation, cell fusion, and Proposed TAM-to-CAF transition. Representative examples are TREM2-positive lipid processing, PAX5-positive CD19-positive TAMs, myeloid-CAF-like hybrid programs, osteoclastogenesis, multinucleated giant-cell formation, and the proposed TAM-to-CAF transition.

### 2.2 A four-level evidence hierarchy for claims of converted identity

Claims are classified by their strongest demonstrated level. Level 1 comprises marker-based observations, including dual immunostaining or co-expression of myeloid and non-myeloid genes. These findings are hypothesis-generating because doublets, ambient RNA, phagocytosed material, membrane transfer, and cell fusion can produce apparent double positivity. Level 2 adds single-cell trajectory, RNA velocity, spatial colocalization, or high-resolution imaging. It supports continuity and localization but does not establish cellular origin or direction. Level 3 requires genetic, inducible, barcode-based, or otherwise validated lineage tracing that identifies the source cell and follows the proposed transition; adoptive transfer is supportive when engraftment artifacts are controlled. Level 4 requires functional demonstration of the proposed converted identity, including a target-lineage regulatory network, stable or reversible state behavior, and a target-cell-specific function. A claim of bona fide transdifferentiation should satisfy the lower levels cumulatively and also document attenuation of the source-lineage program. Evidence can therefore be Level 4 for a specialized macrophage function without demonstrating cross-lineage conversion.

Applied to the tumor-associated macrophage (TAM)–CAF literature, dual-marker and pan-cancer trajectory studies are Level 1–2. Myeloid lineage tracing or adoptive tracing with appropriate controls raises selected organ-specific macrophage-to-myofibroblast transition (MMT) studies to Level 3, and perturbation of SMAD3, RUNX1, or ASCL2 together with matrix-related function provides Level 4 functional support in those models (12–15). Even Level 3–4 evidence does not establish complete conversion in every tumor, because broad myeloid drivers may label multiple progenitors and most human observations remain marker-, trajectory-, or spatially based. TREM2-positive lipid states, osteoclasts, multinucleated giant cells, and vascular-construction macrophages may also have strong functional evidence, but their biological classification remains specialization, intralineage differentiation, fusion, or mimicry rather than transdifferentiation.

## 3 A PU.1-anchored rewritable enhancer model

### 3.1 The myeloid scaffold maintains identity without sealing fate

PU.1/SPI1 is a central lineage-determining factor in macrophage enhancer selection. Together with CCAAT/enhancer-binding proteins (C/EBPs), interferon regulatory factor 8 (IRF8), activator protein 1 (AP-1), RUNX1, and MAF-family proteins, PU.1 establishes accessibility and enhancer marks at selected cis-regulatory elements and creates docking sites for signal-dependent transcription factors. Studies of labelled regulatory elements show that regions occupied or marked by PU.1, IRF8, JUNB, CEBPA, and RUNX1 under resting conditions are differentially deployed during classical or alternative activation (22). Myeloid lineage factors therefore not only stabilize an existing identity but also delimit the genomic regions that can be recruited by future environmental signals.

This scaffold is stable but not closed. Experiments separating cell-intrinsic variation from liver-derived environmental effects show that both components shape the regulatory landscape of Kupffer cells and that transplantation or environmental change remodels a substantial fraction of their epigenomic program (1). In non-alcoholic steatohepatitis (NASH), AP-1 and early growth response factors redirect liver X receptor (LXR)-associated enhancers from resident lipid-homeostatic elements toward injury-response elements. The key change is a change in the local cofactors that collaborate with LXR, allowing recruited monocytes to acquire a TREM2/CD9 lipid-processing and scar-niche state (23). Tumors can also precondition circulating monocytes before they enter a lesion by establishing C/EBP-, AP-1-, and signal transducer and activator of transcription (STAT)-associated chromatin through systemic inflammation (24). Such preconditioning implies that part of the TAM trajectory may be specified in bone marrow or blood. Once the cells enter the tumor, the same signal can then produce a stronger or more sustained output at pre-accessible enhancers, extending the therapeutic window from the tumor itself to circulating monocytes and premetastatic niches.

Disease-associated variants can further modify the threshold at which this scaffold is deployed. A study of an inflammatory bowel disease (IBD)-associated gene desert used genome-wide association mapping, clustered regularly interspaced short palindromic repeat (CRISPR) perturbation, and human functional genomics to identify ETS2 as a major amplifier of macrophage inflammatory and metabolic programs (25). The risk enhancer controls ETS2 abundance, while altered ETS2 dosage coordinately affects inflammatory mediators, reactive oxygen species, glycolysis, and phagocytic genes. Thus, a noncoding risk variant is converted into a disease-scale macrophage state rather than an isolated cytokine change. Genetic susceptibility may therefore set the rewiring threshold, whereas local cytokines and metabolites determine the direction in which that potential is used.

### 3.2 Latent enhancers, signal dynamics, and metabolic licensing

A second basis of macrophage plasticity is the latent enhancer. Different pathogen-associated signals generate distinct NF-κB temporal patterns, including sustained, non-oscillatory, and pulsatile responses. Sustained or non-oscillatory activity more readily opens new chromatin regions and creates lasting, stimulus-specific epigenomic memory (26). SWI/SNF-family complexes then divide the tasks of enhancer selection, chromatin opening, and transcriptional activation (27). Immune stimulation can also remodel enhancer-promoter contacts. For example, NF-κB-dependent looping contributes to programmed death ligand 1 (PD-L1) regulation (28). Locus-specific perturbation shows that these contacts can determine whether selected immune genes are efficiently deployed, rather than merely reflecting ongoing transcription (28).

Metabolic state supplies both the substrates and energetic support for this process. Toll-like receptor (TLR) signaling activates ATP-citrate lyase, increases acetyl-coenzyme A, and promotes histone acetylation, thereby converting metabolic reprogramming into enhancer licensing (29). Interferon-γ (IFN-γ) selectively reinforces STAT1/IRF programs while suppressing a subset of STAT3-associated enhancers (30). Environmental inputs therefore determine more than whether a transcription factor is expressed. They also control when and for how long it accesses chromatin, whether the required metabolic substrates are available, and whether a permissive three-dimensional contact exists.

### 3.3 BACH1 is a plasticity gate rather than a terminal fate determinant

BTB and CNC homology 1 (BACH1) provides a mechanism through which chromatin competence and transcriptional repression coexist. BACH1 occupies open, silent, poised, and latent regulatory regions and interacts with nucleosome remodeling and deacetylase (NuRD) and histone deacetylase complexes. Bach1 loss does not uniformly open chromatin; instead, many regions lose accessibility while a smaller set becomes abnormally accessible, indicating simultaneous roles in chromatin priming and transcriptional repression. After heme, lipopolysaccharide, or interleukin (IL)-4 stimulation, BACH1 redistributes within local chromatin neighborhoods and modifies enhancer–promoter contacts and response thresholds (31).

This mechanism has been termed pioneer repression, but BACH1 does not directly specify whether a macrophage becomes CAF-like, vascular-like, or B-cell-like. BACH1 controls the regulatory reserve available to environmental signals and the threshold for its activation, whereas SMAD, hypoxia-inducible factor, STAT, PPAR/LXR, and other state-specific factors select the terminal direction. During tissue injury and alveolar-macrophage adaptation, BACH1 loss can both dysregulate inflammatory kinetics and prevent normal establishment of repair- and tissue-identity enhancers (31). Its effects are therefore strongly dependent on time, tissue, and ligand context.

### 3.4 IRF8 and BCL6 define identity boundaries and state memory

Compared with BACH1, which mainly controls environmental-response thresholds and chromatin plasticity, IRF8 is more closely aligned with maintenance of mononuclear-phagocyte identity. It collaborates with PU.1 to establish monocyte–macrophage and antigen-presentation programs while limiting granulocytic differentiation and NFATC1-driven osteoclast deviation. In inflammatory arthritis, abnormal methylation of the *Irf8* locus is associated with loss of homeostatic macrophage programs; restoration of Irf8 increases MERTK-, TREM2-, and CX3CR1-associated states and reduces bone erosion (32). In cancer, IRF8 specifies a TAM identity with high major histocompatibility complex (MHC) and antigen-processing capacity, and sustained antigen presentation by this population can promote exhaustion of tumor-specific CD8^+^ T cells (33). IRF8 is therefore neither constitutively pro-inflammatory nor anti-inflammatory; its effect depends on binding partners, antigen persistence, and the local immune context.

B-cell lymphoma 6 (BCL6) is more strongly associated with state stabilization and epigenetic memory. Tumor-derived reduced high-mobility group box 1 (HMGB1) can induce BCL6 through a TLR4–mechanistic target of rapamycin/protein kinase B (mTOR/AKT) pathway. BCL6 then connects TET2, SIRT1, and mitochondrial metabolism, enabling a subset of TAMs to retain self-renewing and tumor-promoting properties after removal from tumor-conditioned medium (34). Existing disease-context evidence, however, is concentrated on candidate loci, metabolism, and cellular function. It has not yet reproduced the genome-wide combination demonstrated for BACH1-occupancy of closed chromatin, bidirectional changes in accessibility, stimulus-dependent redistribution, and three-dimensional genome remodeling. IRF8 and BCL6 should therefore be regarded as candidate dual-function regulators rather than established pioneer repressors.

## 4 Lineage-incongruent states in inflammatory-disease microenvironments

### 4.1 Fibrosis-associated macrophages: from stromal cooperation to myofibroblast-like conversion

Macrophages contribute to chronic fibrosis through three mechanisms. First, they signal to existing fibroblasts by releasing transforming growth factor-β (TGF-β), platelet-derived growth factor, secreted phosphoprotein 1, and metabolites (35–38). In pulmonary fibrosis, a myeloid P2RX4-IL-6-arginase 1 pathway supplies mesenchymal cells with ornithine for collagen synthesis; the macrophage is the metabolic donor, not the collagen-producing cell (35). Spatial analysis of human IBD similarly places inflammatory monocytes and macrophages in defined neighborhoods with fibroblasts and neutrophils (39). These findings show niche cooperation, not macrophage-to-fibroblast conversion. Second, macrophages can acquire a scar-associated transcriptional state. Cross-organ analyses identified CD9^+^TREM2^+^SPP1^+^ macrophages that colocalize with fibroblasts and form a fibrogenic niche (36). Related populations recur in human cirrhosis and muscular dystrophy (37, 38).

The third mechanism comes closer to a true fate conversion. In hypertensive cardiac fibrosis, ALKBH5-dependent N6-methyladenosine regulation of IL-11 was reported to drive acquisition of a myofibroblast program by macrophages (40). In uropathogenic *Escherichia coli* (UPEC)-induced epididymo-orchitis, TGF-β–STAT3 signaling pushed macrophages toward an S100A4^+^, fibronectin 1^high^, collagen-producing state; genetic and pharmacologic interventions reduced fibrosis (17).

Fibrosis-associated markers should not, however, be interpreted as intrinsically pathogenic. Notch2 blockade can induce macrophages expressing SPP1, CD9, GPNMB, and TREM2 while reducing injury and fibrosis in selected lung-damage models (41). Depending on disease stage, this program may enhance lipid and debris uptake, cell survival, and containment of tissue damage. Protection then depends on whether necrotic material is efficiently removed and whether fibroblast stimulation persists. SPP1/TREM2 is therefore understood as a reusable damage-response module than as a context-independent “profibrotic” label.

Evidence further sharpens the boundary between macrophage state change and fate conversion. In rosacea, human proteomics, single-cell and spatial profiling, Cx3cr1-GFP tracing, macrophage depletion, and STAT3-directed intervention support a model-specific macrophage-to-myofibroblast contribution to fibrotic remodeling (42). In contrast, inducible *Lyz2*/*Cx3cr1* tracing and a dual-recombinase MMTracer system found no macrophage contribution to renal myofibroblasts in kidney fibrosis (43). In bleomycin lung injury, genetic tracing instead showed monocyte-derived cells moving through interstitial to alveolar macrophage states, supporting intramyeloid differentiation rather than cross-lineage conversion (44). Together, these studies show that MMT is tissue- and model-dependent and that high-specificity lineage tools can overturn interpretations based on co-expression or broad myeloid labeling.

### 4.2 Lipid-associated macrophages share metabolic modules without becoming adipocytes

Obesity, NASH, and atherosclerosis create lipid-rich environments with dying cells and oxidative stress. A recurring lipid-associated macrophage (LAM) module includes TREM2, CD9, lipoprotein lipase, lysosomal acid lipase, apolipoprotein E, fatty-acid-binding proteins, and galectin 3 (6, 23, 45–49). In obese adipose tissue, TREM2-dependent LAMs surround hypertrophic or dying adipocytes and help buffer lipids (6). CD9^+^ macrophages are enriched for lipid droplets, lysosomes, and inflammatory programs, whereas Ly6C-associated and perivascular populations perform surveillance and clearance (45). This division of labor argues against a single uniform inflammatory state. Time-series and spatial studies suggest a pre-LAM-to-LAM trajectory, but this directionality still rests mainly on trajectory and spatial continuity (46). Across tissues, TREM2 and lipoprotein lipase form a shared lipid-load response, while organ-specific cytokine, matrix, and resident-identity modules remain distinct (47). LAMs from different tissues are therefore not interchangeable.

Long-term obesity can also induce an LY6A^+^ myeloid–mesenchymal hybrid state, which remains conditional evidence rather than established transdifferentiation (18). At the organelle level, lipid droplets, endoplasmic reticulum, mitochondria, and lysosomes form dynamic contact sites that connect fatty-acid storage, membrane-lipid remodeling, oxidation, and lysosomal degradation (48). Disrupting individual contacts or metabolic steps changes inflammatory lipid accumulation, indicating that a lipid-processing identity is also supported by the spatial organization of organelles. Single-cell secretome analysis in NASH places TREM2^+^ macrophages and hepatic stellate cells at central positions in an interaction network: stellate-cell cytokines and chemokines recruit and shape myeloid cells, while macrophage inflammatory and repair ligands feed back to control stellate-cell activation and matrix production (49). The link between lipid-associated states and fibrosis is therefore mediated mainly by bidirectional niche cooperation rather than by macrophages independently executing every stromal task.

TREM2 has context-dependent effects in atherosclerosis. Single-cell and mass-cytometry profiling of human plaques first identified multiple macrophage states associated with symptomatic lesions (50). TREM2 promotes lipid uptake and survival in foam cells (5), and a lipid-associated state can transition toward an inflammatory state associated with cerebrovascular complications (51). Conversely, TREM2-mediated phagocytosis and survival can limit formation of the necrotic core (52). Early intermittent hyperlipidemia can also imprint a persistent macrophage memory that amplifies later atherosclerosis even when lipid exposure is discontinuous (53). Soluble TREM2 and TREM2^+^ macrophages in NASH may similarly reflect a protective response that accumulates within fibrotic regions and limits injury (54).

Lipid-associated macrophages do not become adipocytes. They deploy lipid-uptake, lipid-droplet, lysosomal, and cholesterol-efflux modules shared with adipose and foam-cell biology. Their pathogenic effect cannot be inferred from TREM2 or SPP1 alone; it depends on lipid composition, whether clearance capacity is saturated, and the disease stage.

### 4.3 Vascular-like functions: channel construction versus perivascular cooperation

Macrophages usually support angiogenesis by releasing vascular endothelial growth factor A (VEGFA), degrading matrix, guiding endothelial sprouts, and occupying perivascular positions (8, 9, 16, 55, 56); none of these functions alone means that the cell has become a mature endothelial cell. Zebrafish fin regeneration provides more direct evidence of functional mimicry: macrophages at the injury front align, elongate, and enclose lumen-like structures connected to existing vessels, and perfusion tracing shows that some channels enter the circulation (16). Electron microscopy and depletion experiments support direct macrophage participation in channel construction rather than simple adhesion to an endothelial surface. Nevertheless, the ultrastructure and marker network differ from mature endothelial vessels, making transient vascular-construction mimicry the more accurate definition.

Perivascular niches can also stabilize a specialized identity without endothelial transdifferentiation. c-MAF-dependent LYVE1/FOLR2/CD38-associated perivascular macrophages participate in diet-induced metabolic syndrome. This finding indicates that c-MAF converts signals from the vascular niche into a persistent perivascular macrophage program that influences vascular homeostasis and metabolic inflammation (8). Brioschi et al. showed that c-MAF drives insulin-like growth factor-1 (IGF1) expression in brain perivascular macrophages, enabling communication with endothelial cells. Conditional c-MAF deletion altered cerebral vascular function in mice, while human macrophages shared the regulatory module; this supports macrophage–endothelial cooperation, not endothelial conversion (57).

Stringent evidence for endothelial transdifferentiation would require coordinated activation of a mature endothelial regulatory network-including ERG, ETV2, SOX17, TAL1, KDR, CDH5, and ESAM-together with polarity, basement-membrane organization, and barrier function (16, 56). Current evidence therefore supports macrophage-mediated vascular mimicry or vascular-construction mimicry rather than a general macrophage-to-endothelial conversion.

### 4.4 Osteoclasts and multinucleated giant cells: intralineage differentiation and fusion

Osteoclasts arise from the monocyte–macrophage lineage. Osteoclastogenesis is therefore one of the most robust examples of macrophage plasticity but is not a cross-lineage conversion. RANKL–RANK signaling activates NF-κB and c-FOS, induces positive feedback of nuclear factor of activated T cells 1 (NFATC1), and cooperates with PU.1, AP-1, and MITF to establish bone-resorptive and fusion genes, including ACP5, CTSK, MMP9, DCSTAMP, and ATP6V0D2 (19). In human and mouse RANKL-induced osteoclastogenesis and inflammatory arthritis, RANKL establishes osteoclast-specific super-enhancers and conventional enhancers early in differentiation and recruits RNA polymerase II to generate enhancer RNAs (19). Interference with selected enhancer RNAs reduces expression of nearby osteoclast genes and impairs bone resorption, demonstrating that enhancer transcription is a functional step that advances the macrophage scaffold toward an NFATC1-dependent terminal fate.

Non-osteoclastic multinucleated giant cells (MGCs) arise mainly by cell fusion. IL-4/IL-13-STAT6 signaling establishes fusion competence, and E-cadherin/catenin complexes support cell contact. DC-STAMP is required for fusion of osteoclasts and foreign-body giant cells. β-catenin has context-dependent effects; in selected IL-4 models, myeloid β-catenin deletion increases multinucleation (58–60). Human studies show that DC-STAMP knockdown reduces fusion. Multinucleation also suppresses parts of the mononuclear-phagocyte program, including CSF1R, MRC1, CD163, TLR2, MAFB, FOS, and JUNB, and produces distinct immune, phagocytic, or mitochondrial functions (20). Fusion is therefore a functional reset with remodeling of macrophage-associated programs, not simply an increase in cell size. Different MGC classes should not be treated as equivalent. In liver fibrosis, recruited monocytes form CD36-dependent Kupffer cell-like syncytia expressing Kupffer-cell markers and displaying enhanced bacterial capture; related syncytia were also observed in human cirrhosis (61). During allergen-induced type 2 lung inflammation, an IL-33–ILC2–IL-13 pathway induces IRF4, which suppresses PPARγ expression and dismantles the resident-alveolar homeostatic regulon while activating chemokine-production and cell-fusion programs (4). Xue et al. identified an additional fusion mechanism in colorectal cancer after neoadjuvant chemotherapy: apoptotic cell overload activates TREM2/TYROBP–MAPK/c-FOS signaling to increase DC-STAMP transcription, and TYROBP knockdown or loss inhibits macrophage fusion (62). These findings demonstrate macrophage state remodeling and fusion without establishing cross-lineage transdifferentiation.

### 4.5 Synovial macrophages: IRF8 links identity homeostasis to the osteoclast boundary

Inflammatory arthritis combines monocyte recruitment, remodeling of synovial macrophages, and osteoclast differentiation. Aberrant methylation and reduced expression of Irf8 can promote loss of homeostatic programs and an osteoclast bias. Low-dose demethylating treatment restores Irf8, expands macrophage states associated with MERTK, TREM2, and CX3CR1, and reduces inflammation and bone erosion (32). These data support restoration of an identity boundary as a therapeutic concept, although demethylating agents affect multiple cell types and the benefit cannot yet be assigned exclusively to synovial macrophages (**Table 1**).

**Table 1:** Lineage-incongruent and related macrophage states in inflammatory-disease microenvironments.

Disease or niche	Acquired macrophage module	Principal signal or transcription factor	Evidence level and strength	Most defensible biological definition	Representative references
Fibrosis of lung, liver, or skeletal muscle	SPP1/TREM2/CD9; matrix organization and fibroblast cooperation	GM-CSF, IL-17A, TGF-β1, ARG1	Levels 2 and 4 for niche localization and function; no direct fibroblast fate tracing	Profibrotic niche state; usually not direct fibroblast transdifferentiation	(35–38)
Cardiac or post-infectious fibrosis	S100A4, FN1, collagen, and myofibroblast programs	ALKBH5–IL-11; TGF-β–STAT3	Levels 2–4 in model systems; origin tracing and source-program loss remain incomplete	MMT candidate; requires more specific tracing of mature macrophages	(17, 40)
Obese adipose tissue	TREM2/LPL/LIPA/APOE; lipid droplets and crown-like structures	TREM2, local lipids, and lysosomal networks	Levels 2–4 for lipid specialization; no adipocyte conversion	Lipid-processing specialization, not adipocyte transdifferentiation	(6, 18, 45, 46)
NASH and liver fibrosis	TREM2/CD9 lipid–injury module and stellate-cell communication	AP-1/EGR redirection of LXR; stellakine network	Levels 2–4 for niche reprogramming and stromal cooperation; no lineage conversion	Liver-niche reprogramming and stromal cooperation	(23, 49, 54, 84)
Atherosclerosis	Foam-cell formation, lipid uptake, survival, or limitation of necrosis	TREM2, TLRs, and intermittent-hyperlipidemia memory	Levels 2–4 for state and function; disease-stage effects conflict	Disease-stage-dependent lipid state	(5, 50–53)
Regeneration and perivascular niches	Perfusable channels or LYVE1/FOLR2-associated perivascular function	Regenerative/hypoxic signals; c-MAF–IGF1	Level 4 functional mimicry by perfusion/depletion or c-MAF perturbation; no endothelial fate	Vascular-construction mimicry or perivascular specialization; not mature endothelial conversion	(8, 16, 57)
Inflammatory arthritis	Bone resorption, fusion, and homeostatic-to-osteoclast branching	RANKL–NF-κB–c-FOS–NFATC1 and IRF8	Level 4 functional evidence for intralineage osteoclast differentiation	Osteoclast terminal differentiation within the myeloid lineage	(19, 32)
Granuloma and foreign-body responses	Langhans-type or foreign-body MGC formation and functional resetting	IL-4/IL-13–STAT6, E-cadherin, and DC-STAMP	Level 4 fusion and functional evidence; not cross-lineage conversion	Macrophage fusion and multinucleation, not cross-lineage conversion	(20, 58–60)

## 5 Lineage-incongruent states and functional takeover in the tumor microenvironment

Pan-cancer single-cell atlases show that TAMs comprise conserved states shared across tumor types and organ-specific modules; their relationship to immunotherapy cannot be reduced to a single M1/M2 axis (63). The CAF-like, vascular-like, B-lineage-like, metabolic, and multinucleated states discussed below are intersecting functional dimensions rather than fixed lineages. Competing interpretations must remain explicit: non-myeloid gene expression may reflect activation or material exchange, trajectories do not establish fate, broad myeloid tracing tools may label heterogeneous progenitors, and causal experiments are concentrated in mouse or transplantable models. The evidence levels assigned in **Table 2** therefore describe experimental support, not an automatic diagnosis of lineage conversion. An updated pan-cancer analysis integrating 1.39 million single cells, spatial transcriptomics, and bulk cohorts identified convergent inflammatory endpoints in tissue-resident and monocyte-derived macrophages (64). This supports strong environmental remodeling of distinct macrophage origins, but the reliance on trajectory and state convergence does not by itself establish cross-lineage fate conversion.

**Table 2:** Evidence levels, lineage-incongruent macrophage states, and biological boundaries in tumor microenvironments.

Tumor-associated state	Acquired module	Environmental input and regulatory axis	Functional consequence	Evidence level and boundary	Representative references
Collagen-producing TAM	Collagen synthesis, ECM stiffening, and CAF-like matrix	TGF-β and metabolic reprogramming	Restricted CD8+ T-cell activity and metabolic suppression	Level 4 macrophage matrix function; not a complete CAF fate	(7)
SPP1+ TAM–CAF network	ECM organization, proximity to FAP+ CAFs, and immune exclusion	Hypoxia, TGF-β/IL-1, and spatial interaction	Immunosuppressive stromal network	Level 2 spatial cooperation; no evidence of cellular-origin conversion	(65, 66)
MMT and macrophage-derived CAF	ACTA2/FAP/FN1/collagen program	TGF-β1–SMAD3–RUNX1	Increased CAF abundance and tumor progression	Levels 3–4 in selected models; incomplete source-program loss and limited causal human evidence	(12, 13)
Bone-metastatic CAF_7	MHC-II/myeloid traces plus a myofibroblast program	ASCL2–IL6RA	More Tregs, CD8 exhaustion, and PD-1 resistance	Levels 3–4 in an organ-specific model; requires independent and human causal replication	(14, 15)
Vascular-associated TAM	Perivascular localization, angiogenesis, or competition with endothelial progenitors	Hypoxia, TSC1–mTORC1, and VEGFA/Flt-1	Promotion or inhibition of neovascularization	Levels 2–4 for vascular function or competition; no mature endothelial identity	(9, 55, 56, 68)
Iron-rich EDNRB+ iTAM	Iron processing, angiogenesis, and immunosuppression	Heme–BACH1 degradation–EDNRB	Increased vascular density and tumor growth	Level 4 state function; no mature endothelial identity	(10)
CD19+ B-lineage-like TAM	CD19, OXPHOS, PD-L1/CD73, and lysosomal insufficiency	PAX5-associated program	Immunosuppression and CAR-T targetability	Levels 1–4 for state and animal targetability; stable macrophage-intrinsic CD19 remains unresolved	(11)
B-cell precursor to macrophage-like cell	Myeloid phagocytosis and macrophage markers	Tumor-derived M-CSF/CSF1R and PAX5 downregulation	Expansion of the tumor myeloid ecosystem	Levels 3–4 for the reverse B-cell-to-myeloid direction; not macrophage-to-B-cell conversion	(69)
Lipid/metabolic TAM	TREM2, FABP/LPL, XBP1, SREBP1, and lactate or purine metabolism	Lipids, lactate, PARP therapy, and tumor-derived metabolites	Immunosuppression, survival, metastasis, and resistance	Levels 2–4 for metabolic specialization; not adipocyte or tumor-cell transdifferentiation	(70–78)
IRF8+ or BCL6+ TAM	Antigen-presentation/exhaustion or stable memory-like state	IRF8; HMGB1–TLR4–mTOR–BCL6	T-cell exhaustion or durable protumor memory	Levels 2–4 for regulatory state and function; incomplete genome-wide causal mechanism	(33, 34)
Tumor-associated MGCs (PDAC and colorectal cancer)	PDAC: stress/MYC programs; colorectal cancer: efferocytosis-induced fusion	PDAC axis unresolved; colorectal cancer: TREM2/TYROBP–MAPK/c-FOS–DC-STAMP	PDAC: aggressive niches; colorectal cancer: favorable regression and preclinical tumor suppression	PDAC: Levels 1–2; colorectal cancer: human localization plus causal fusion and antitumor function in models; no cross-lineage conversion	(80, 62)

### 5.1 The TAM–CAF continuum: the strongest candidate, but not a general proof of conversion

Evidence linking TAMs to CAFs spans a gradient from functional cooperation to candidate conversion. In breast cancer, TAMs directly deposit collagen, increase matrix density, and restrict CD8^+^ T-cell function (7). This is Level 4 evidence for a macrophage-executed matrix function, not evidence that the cell has become a CAF. In colorectal cancer and colorectal liver metastases, SPP1^+^ TAMs form spatially adjacent, immunosuppressive networks with fibroblast activation protein-positive CAFs (65, 66). These Level 2 studies establish niche organization and cooperation but do not show conversion of cellular origin.

Organ-specific macrophage-myofibroblast transition (MMT) studies provide stronger evidence for fate conversion. CD68^+^ACTA2^+^ transitional cells are detectable in NSCLC and mouse tumors. Myeloid lineage tracing, adoptive transfer, pseudotime, RNA velocity, *in vitro* induction, and macrophage-restricted Smad3 deletion support macrophage origin and partial acquisition of a CAF-like program (12). SMAD3 binds regulatory regions linked to differentiation and matrix production. Its pharmacologic inhibition or genetic deletion reduces transitional cells, CAF abundance, and tumor growth (12). RUNX1 acts downstream of TGF-β1-SMAD3 and links myeloid chromatin to ACTA2, FAP, fibronectin 1, and collagen programs; deletion or Ro5–3335 treatment reduces MMT (13). These experiments provide Level 3–4 support in the tested models. They do not establish general TAM-to-CAF conversion because lineage-tool specificity, incomplete measurement of source-program loss, and limited causal testing in human tumors remain unresolved.

In NSCLC bone metastases, single-cell analyses identified a CAF_7 population coexpressing CD68, CD163, MRC1, CD74, and HLA-DRA with ACTA2, FAP, COL1A1, and COL1A2 (14). Endogenous and adoptive lineage tracing and perturbation provide Level 3–4 support for a TAM-to-CAF_7 direction in this organ-specific model and identify ASCL2-dependent IL6RA activation. Silencing *ASCL2* or *Il6ra* reduces CAF_7 cells, regulatory T cells, and CD8^+^ T-cell exhaustion while enhancing PD-1 blockade (14). A pan-cancer analysis proposed a TAM-to-antigen-presenting CAF-to-myofibroblastic CAF trajectory along which SPI1 and MAFB decrease while ACTA2 and MYLK increase (15), but this remains Level 2 support. It does not replace organ-specific tracing, and it cannot exclude convergence of independently derived populations on a similar transcriptional state.

A 2026 bladder-cancer study further nominated KLF6 as a driver of an ACTA2^+^ macrophage-to-myofibroblast program associated with PD-L1 expression and immunotherapy resistance (67). Spatial pseudotime, KLF6 overexpression, virtual knockout, and *in vivo* tumor experiments support a functional MMT-like state, but the absence of macrophage-specific genetic fate mapping places this study below definitive evidence for stable cellular-origin conversion.

Within the candidate hierarchy (TGF-β1→SMAD3→RUNX1 or organ-specific ASCL2–IL6RA→extracellular-matrix and myofibroblast programs.), SMAD3 acts as an environmental signal decoder; RUNX1 and ASCL2 act as fate-bridging or state-stabilizing regulators; and reduced SPI1/MAFB activity is consistent with attenuation of myeloid identity. Direct longitudinal demonstration of all three events in the same human-derived cells remains lacking.

### 5.2 The vascular niche: angiogenesis, cellular competition, and vascular mimicry

Pan-cancer myeloid atlases show that angiogenic TAMs are not identical across cancers but are shaped by the organ, tumor type, and local hypoxia (55). In colorectal liver metastasis, F4/80^+^ TAMs accumulate around new vessels and promote vascularization (9). This demonstrates proangiogenic activity and spatial positioning rather than a mature endothelial identity. Reprogrammed TAMs can also compete with endovascular endothelial progenitor cells and suppress tumor neoangiogenesis (56), showing that macrophages can regulate a vascular niche through cellular competition without becoming endothelial cells. It is also essential to distinguish macrophage-constructed channels from macrophage-induced tumor-cell vascular mimicry. Metadherin-high macrophages promote VEGFA-165/Flt-1-dependent vascular mimicry by head and neck squamous carcinoma cells, but the cancer cell-not the macrophage-is the mimetic cell (68).

Tumor hemorrhage and heme generate a distinct iron-rich, endothelin receptor type B-positive TAM state (EDNRB^+^ iTAM). Heme promotes BACH1 degradation and releases the associated state program; these macrophages occupy perivascular and stromal regions and perform angiogenic and immunosuppressive functions (10). Myeloid *Ednrb* deletion reduces vascular density and tumor growth, and endothelin-receptor blockade cooperates with PD-1 blockade (10). This work links a metabolic input, a chromatin gate, and vascular function but does not demonstrate conversion of an iTAM into an endothelial cell.

### 5.3 B-lineage-like boundary states and reverse lineage co-option

CD19^+^ TAMs described in hepatocellular carcinoma (HCC) retain CD14, CD68, and other myeloid features while acquiring a PAX5-associated program, increased mitochondrial oxidation, lysosomal insufficiency, and accumulation of PD-L1 and CD73. PAX5 perturbation alters this immunosuppressive state, and anti-CD19 chimeric antigen receptor T-cell (CAR-T) therapy exploits the newly acquired surface antigen to suppress tumors in animal models (11). CD19 accumulation does not represent complete B-cell differentiation; it is more accurately described as a PAX5-driven, B-lineage-program-borrowing TAM state.

Tumors can co-opt the same lineage boundary in the opposite direction. Tumor-derived macrophage colony-stimulating factor/colony-stimulating factor 1 receptor (M-CSF/CSF1R) signaling causes B-cell precursors to downregulate PAX5 and differentiate into macrophage-like cells (69). This finding demonstrates bidirectional plasticity at the B-cell–myeloid boundary but does not show that a mature macrophage becomes a B cell. Considering both directions highlights that PAX5 is not a simple binary B-cell switch; its effect depends on the starting cell and the surrounding regulatory network.

### 5.4 Lipid- and metabolically specialized TAMs: functional convergence rather than morphological transdifferentiation

Glucose, lipid, lactate, purine, and redox stresses in the tumor microenvironment generate highly specialized metabolic TAM states. Genetic loss or antibody targeting of TREM2 remodels the immunosuppressive myeloid compartment and enhances PD-1 blockade (70, 71). TAMs associated with lung metastases or the tumor–adipose boundary express LGALS3/TREM2 or FABP3/4/5-LPL-LIPA programs that combine lipid processing, extracellular-matrix remodeling, and immune suppression (72, 73).

Tumor-derived glucosylceramide reshapes endoplasmic-reticulum membrane lipids in TAMs, activates an inositol-requiring enzyme 1—X-box binding protein 1—STAT3 pathway, and supports protumor macrophage survival (74). Human tumor-conditioned media likewise increase macrophage synthesis of fatty acids, phospholipids, and sphingolipids (75). A terminal purine-metabolic TAM state develops in liver metastases (76), while lactate-rich conditions create heterogeneity in lactate utilization, arginine metabolism, and immunosuppression (77). Following poly (ADP-ribose) polymerase (PARP) inhibition in BRCA1-associated triple-negative breast cancer, sterol regulatory element-binding protein 1-driven macrophages acquire combined glycolipid-metabolic and immunosuppressive features that contribute to resistance (78).

These macrophages share selected metabolic programs with adipocytes, tumor cells, and other metabolically active cell types but do not acquire their full lineage identities. Their importance also illustrates that mimicry can take the form of functional competition: metabolic reprogramming can enable macrophages to outcompete cancer cells for nutrients and increase phagocytosis (79), thereby redirecting a normally protumor adaptation toward an antitumor function.

### 5.5 Tumor-associated multinucleated giant cells

In human pancreatic ductal adenocarcinoma (PDAC), CD68^+^ MGCs constitute a distinct macrophage population enriched in squamous, non-glandular regions and in tumors exposed to neoadjuvant treatment. They exhibit DNA-damage, oxidative-stress, and MYC-related programs and are associated with an aggressive microenvironment (80). This study establishes the presence of macrophage-derived MGCs in human cancer but does not identify STAT6, NFATC1, or DC-STAMP as their direct upstream driver in PDAC. By contrast, Xue et al. observed CD68-positive MGCs in apoptotic-cell-rich regions of colorectal tumors showing favorable regression after neoadjuvant chemotherapy. Experimentally generated efferocytosis-induced MGCs inhibited colorectal cancer cell growth and suppressed tumor growth in mice, with fewer CD206-positive macrophages and regulatory T cells and greater CD8-positive T-cell infiltration (62). Thus, tumor-associated MGCs can have context-dependent, opposing functions; these preclinical findings do not establish clinical benefit or cross-lineage conversion.

Many reported tumor-associated macrophage states may reflect either partial regulatory conversion or activation of reusable macrophage programs in response to tumor-derived cytokines, metabolites, hypoxia, phagocytosis, or membrane exchange (**Table 2**). Thus, extracellular-matrix, vascular, or B-lineage markers alone do not establish fate conversion and require orthogonal exclusion of doublets, ambient RNA, transferred proteins, and fusion. Of the 11 tumor-state categories in **Table 2**, only macrophage-to-myofibroblast transition (MMT)/macrophage-derived CAF and bone-metastatic CAF_7 currently include direct lineage- or origin-tracing evidence for a TAM-to-CAF direction; even these data are concentrated in mouse, transplantable, or organ-specific models, whereas most human evidence remains at Level 1-2. Other categories more strongly support specialization, niche cooperation, reverse B-cell-to-myeloid co-option, metabolic adaptation, or fusion.

## 6 A heuristic mechanistic framework: how the environment may select target modules on a myeloid scaffold

Evidence from inflammatory disease and cancer suggests a four-component heuristic framework. It is an organizing hypothesis, not a validated rule for determining macrophage identity. The components overlap, may operate in different sequences, and may not apply in every tissue or disease stage. Alternative regulatory architectures remain possible.

The first proposed component is a myeloid identity scaffold built by PU.1, C/EBPs, IRF8, MAFB/c-MAF, RUNX1, and related factors. It supports core myeloid functions and shapes much of the accessible enhancer landscape. A second, overlapping component is a plasticity gate that includes primed and latent enhancers, BACH1-NuRD, SWI/SNF complexes, NF-κB dynamics, metabolic histone modification, and three-dimensional chromatin organization. A third component comprises context-dependent signal decoders, including TGF-β-SMAD3, hypoxia/heme/HIF-BACH1-EDNRB, IL-4/IL-13-STAT6, IFN-STAT, PPAR/LXR/SREBP/XBP1, and RANKL-NF-κB-c-FOS-NFATC1 pathways. A fourth component includes candidate state-bridging or state-locking factors such as RUNX1, ASCL2, PAX5, and BCL6. Evidence for these components varies substantially by macrophage state. Their grouping does not show that four components are necessary or sufficient to determine identity. The model can be expressed as: Retention of a myeloid core + context-selective deployment of a noncanonical functional module = a lineage-incongruent macrophage state, not necessarily a lineage transition.

When most of the myeloid core is retained, the outcome is usually functional mimicry or a hybrid state. A state approaches transdifferentiation only when the source-lineage network is progressively attenuated, a target-lineage regulatory network is stably reinforced, and the corresponding function is acquired. This framework may also explain why the same markers have different effects across diseases. TREM2, SPP1, CD9, and GPNMB participate in reusable modules for lipid uptake, lysosomal adaptation, survival, matrix organization, and damage clearance. These modules can limit necrosis when lipids are safely sequestered and cleared (52, 54). Under persistent stimulation, matrix feedback, or tumor immunosuppression, they can instead support fibrosis and tumor progression (36, 65, 66, 70–72). Functional prediction therefore requires joint consideration of marker combinations, ontogeny, spatial position, disease stage, and downstream effects.

## 7 Translational implications: distinguishing hypotheses from clinically actionable strategies

We separate therapeutic proposals into four maturity tiers (**Table 3**): mechanistic target hypotheses, preclinical proof of concept, early clinical evidence, and clinically established myeloid-niche therapies. A lineage-incongruent marker is not clinically actionable unless its cell-intrinsic origin and stability, functional relevance, selectivity, safety window, companion biomarker, and efficacy are demonstrated in appropriate human studies. **Table 3** uses these tiers to prevent extrapolation from animal perturbation to clinical recommendation.

**Table 3:** Therapeutic strategies targeting lineage-incongruent macrophage states, classified by translational maturity.

Therapeutic level	Target state or molecule	Representative intervention	Maturity tier	Main result	Principal risk and development need	Representative references
Use a borrowed surface antigen	CD19+ B-lineage-like TAM/CD19	Anti-CD19 CAR-T	Preclinical proof of concept	Suppressed HCC growth	Also depletes B cells; cytokine-release and macrophage-activation risks require evaluation	(11)
Block a state-specific effector receptor	Iron-rich EDNRB+ iTAM/EDNRB	Macitentan plus PD-1 blockade	Preclinical mechanism/combination	Reduced vascular density and tumor burden	Macitentan is not EDNRB- or TAM-specific; heme/EDNRB companion diagnostics are needed	(10)
Subset-directed antibody	TREM2+ TAM/TREM2	PY314 with or without pembrolizumab	Early clinical; limited activity	Feasible with limited clinical activity	Small, heavily pretreated, nonrandomized cohort; TREM2 can be protective in other organs	(52, 54, 81)
Block an upstream MMT pathway	MMT/SMAD3	SIS3 or macrophage Smad3 deletion	Preclinical validation	Reduced MMT, CAFs, and tumor growth	Systemic TGF-β/SMAD3 inhibition has a narrow homeostatic window	(12)
Block a fate-bridging factor	MMT/RUNX1	Ro5–3335 or Runx1 deletion	Preclinical validation	Reduced F4/80+α-SMA+ transitional cells	Neither the drug nor the transcription factor is macrophage-specific; targeted delivery is required	(13)
Block an organ-specific fate	Bone-metastatic CAF_7/ASCL2–IL6RA	ASCL2 or Il6ra silencing plus PD-1 blockade	Preclinical organ-specific validation	Reduced CAF_7, Tregs, and CD8 exhaustion	Single organ-specific report; no mature selective ASCL2 drug	(14)
Restore an identity boundary	Arthritic macrophage/IRF8 methylation	Low-dose demethylation or Irf8 restoration	Preclinical multi-model validation	Restored homeostatic macrophages and reduced bone erosion	Demethylation affects many cells; synovial-macrophage-targeted delivery is needed	(32)
Exploit a transcription-factor-created dependency	PAX5-associated TAM/CD73 or OXPHOS	CD73 or metabolic inhibition plus checkpoint blockade	Preclinical proof of concept	Improved checkpoint therapy	Metabolic targets lack cellular specificity	(11)
Stratify rather than systemically inhibit	BACH1-low/EDNRB-high iTAM	Stratify by BACH1/heme and target EDNRB	Mechanistic hypothesis	Avoids induction of angiogenic states by BACH1 inhibition	BACH1 also maintains normal inflammatory thresholds and tissue adaptation	(10, 31)
Treat a mature pathological myeloid niche	Tenosynovial giant cell tumor/CSF1R	Vimseltinib	Clinically established (phase III)	Improved tumor and functional outcomes	Shows myeloid-niche druggability but does not validate a TAM transdifferentiation therapy	(82)
Counterexample to broad depletion	CSF1-dependent TAMs in solid tumors/CSF1R	Pexidartinib plus durvalumab	Early clinical/mechanistic	Limited efficacy and disruption of FLT3-dependent dendritic cells	Supports state-selective intervention over broad macrophage depletion	(83)

### 7.1 Targeting surface molecules acquired by abnormal states

A newly detected surface molecule can provide an experimental entry point. Anti-CD19 CAR-T cells eliminate CD19^+^ TAMs and suppress HCC in animal models (11), providing preclinical proof of concept rather than a clinically actionable macrophage therapy. It remains unresolved whether CD19 in these TAMs reflects stable cell-intrinsic transcriptional reprogramming in all settings, transient protein acquisition, phagocytosed material, membrane transfer, or tumor-associated molecular exchange. Translation would additionally require validation of macrophage-intrinsic CD19, persistence over time, functional dependence, and assessment of B-cell depletion, cytokine-release syndrome, and macrophage-activation risks.

Iron-loaded EDNRB^+^ iTAMs provide a second preclinical strategy. Targeting the downstream EDNRB-associated state may be more selective than perturbing BACH1, which has broad roles in chromatin adaptation; macitentan combined with PD-1 blockade is supported by animal data (10). TREM2-directed antibodies have entered early clinical testing. PY314 alone or with pembrolizumab in platinum-resistant ovarian cancer established feasibility but showed limited activity in a small, heavily pretreated, nonrandomized cohort (81). TREM2 also supports lipid clearance, tissue repair, survival, or limitation of necrosis in several physiological and disease contexts (6, 52, 54). Systemic depletion may therefore remove protective macrophages, and neither the EDNRB nor TREM2 approach should be described as clinically actionable without tissue-specific biomarkers, controlled efficacy data, and organ-safety assessment.

### 7.2 Blocking formation or maintenance of a pathogenic fate

The TAM-to-CAF axis provides a relatively complete preclinical intervention chain. SMAD3 inhibition or macrophage-specific *Smad3* deletion reduces MMT and tumor growth (12). Ro5-3335-mediated RUNX1 inhibition and *Runx1* deletion reduce F4/80^+^α-smooth muscle actin-positive transitional cells (13). In bone metastasis, silencing *ASCL2* or *IL6RA* reduces CAF_7 cells, Tregs, and CD8+ T-cell exhaustion and improves PD-1 blockade (14). From a therapeutic-window perspective, state-specific surface or effector targets are likely to be preferable, followed by fate-bridging factors such as RUNX1 or ASCL2, with broad TGF-β/SMAD3 inhibition as a less selective option. TGF-β/SMAD3 participates in immune tolerance, normal repair, and homeostasis across many cell types, making systemic blockade intrinsically costly.

PAX5, IRF8, and BCL6 also have therapeutic potential but may be better approached indirectly or through targeted delivery. PAX5-associated TAMs could be attacked through CD19, CD73, or oxidative-phosphorylation dependencies rather than by direct PAX5 inhibition. Low-dose demethylating therapy restores Irf8 and improves experimental arthritis, supporting therapeutic restoration of an identity boundary (32), although the drugs act on many cell types. BCL6 is required for formation of memory-like protumor TAMs (34), but macrophage-targeted clinical candidates have not yet been developed.

### 7.3 The directional hazard of targeting BACH1

BACH1 should not be treated as a target for which lower activity is invariably beneficial. BACH1 maintains enhancer priming, inflammatory-response thresholds, tissue adaptation, and damage repair (31). Conversely, heme released during tumor hemorrhage promotes BACH1 degradation and releases an EDNRB-associated angiogenic iTAM program; *Bach1* loss can therefore aggravate this abnormal state (10). Systemic BACH1 inhibition could enhance selected immunosuppressive and angiogenic TAM programs. A more rational strategy is to use BACH1 activity or heme burden as a niche-stratification marker and target state-specific downstream effectors such as EDNRB, or to develop spatiotemporally controlled macrophage-delivery systems.

### 7.4 Clinically mature myeloid-niche therapies and the limitations of broad depletion

Therapies targeting RANKL and CSF1R demonstrate that pathological monocyte–macrophage lineage niches are clinically druggable. RANKL inhibition blocks osteoclast formation and activity and is used for giant cell tumor of bone and skeletal complications. In CSF-1-driven tenosynovial giant cell tumor, the randomized, double-blind phase III MOTION trial showed that vimseltinib improves both tumor and functional outcomes (82).

Broad TAM depletion may remove beneficial myeloid populations. In advanced solid tumors, pexidartinib affects CSF1R-dependent myeloid cells, disrupts FLT3-dependent dendritic-cell differentiation, and may antagonize durvalumab (83). Among the examples in **Table 3**, randomized phase III efficacy is established for vimseltinib in tenosynovial giant cell tumor, a mature CSF1-driven myeloid niche (82); this does not validate therapies directed at putative TAM lineage conversion. CD19-, EDNRB-, TREM2-, SMAD3-, RUNX1-, ASCL2-, and PAX5-associated strategies remain mechanistic, preclinical, or early clinical. Therapeutic development should therefore prioritize state selectivity while preserving antigen presentation, phagocytosis, lipid clearance, and tissue repair.

## 8 Discussion and outlook

Several major questions remain unresolved. First, is there a universal plasticity switch shared by all lineage-incongruent macrophage states? BACH1 provides a pioneer-repression model, but there is no evidence that CAF-like, vascular-like, B-lineage-like, and osteoclast states depend on the same BACH1-bound elements. Whether IRF8, BCL6, c-MAF, or other repressive transcription factors similarly combine chromatin priming with transcriptional restraint will require acute protein degradation, nucleosome-binding assays, genome-wide occupancy mapping, and three-dimensional chromatin analysis.

Second, how does one TREM2^+^SPP1^+^ or CD9^+^ starting population diverge into alternative fates? In bone metastasis or chronic inflammation, SMAD3—RUNX1/ASCL2, RANKL—NFATC1, and IL-4/IL-13—STAT6 may promote CAF-like, osteoclast, and non-osteoclastic MGC states, respectively. Whether these networks compete for the same PU.1-primed enhancers or form mutually inhibitory fate switches is a key experimental question.

Third, do tissue-resident and monocyte-derived macrophages have different capacities for state remodeling and hybrid-program acquisition? Loss of resident macrophages, replacement by recruited monocytes, and local reprogramming can converge on similar endpoints in NASH (84). Peripheral monocytes can also be epigenetically preconditioned before entering a tumor (24). Future experiments should trace embryonically derived resident macrophages and bone-marrow-derived monocytes simultaneously within the same model.

Fourth, do lineage-incongruent states generate therapeutic memory? BCL6-associated TAMs and early intermittent hyperlipidemia both show that environmental exposure can leave a durable state (34, 53). Chemotherapy, radiotherapy, antiangiogenic therapy, and immune-checkpoint blockade may therefore do more than select pre-existing subsets; they may actively generate new CAF-like, MGC-like, or metabolically specialized macrophages.

Fifth, how can state-selective delivery be made safe? An ideal intervention would require convergence of a surface target, a spatial niche, and a regulatory dependence-for example, delivering ASCL2/IL6RA interference selectively to bone metastases or using endothelin-receptor blockade only in heme-high tumors enriched for EDNRB^+^ TAMs. Single-cell and spatial atlases must progress from subset discovery toward companion diagnostics, patient stratification, and pharmacodynamic monitoring.

Macrophages cannot become any cell type without constraint. The explanation most consistent with current evidence is that they often retain a myeloid regulatory scaffold while deploying context-selected functional modules. The proposed PU.1-anchored enhancer architecture offers a testable mechanism for this behavior. Physiological specialization, osteoclastogenesis, and fusion alone do not establish cross-lineage conversion. Only a minority of organ-specific TAM-to-CAF reports currently approach stable cross-lineage conversion, and causal evidence in human tumors remains limited.

This boundary-aware framework compares fibrosis, lipid handling, vascular construction, antigen presentation, osteoclastogenesis, and cell fusion without treating them as equivalent lineage events. It also supports a cautious translational sequence: validate the state and its evidence level, establish target stability and function, demonstrate selective efficacy and safety in models, and only then test clinical actionability. The field will advance through origin-resolved, same-cell, longitudinal evidence.

## Glossary

AP-1: activator protein 1
ATAC: assay for transposase-accessible chromatin
BACH1: BTB and CNC homology 1
BAF: BRG1/BRM-associated factor
BCL6: B-cell lymphoma 6
CAF: cancer-associated fibroblast
CAR-T: chimeric antigen receptor T cell
C/EBP: CCAAT/enhancer-binding protein
ChIP-seq: chromatin immunoprecipitation sequencing
CRISPR: clustered regularly interspaced short palindromic repeats
CSF1R: colony-stimulating factor 1 receptor
DC: dendritic cell
DC-STAMP: dendritic cell-specific transmembrane protein
ECM: extracellular matrix
EDNRB: endothelin receptor type B
FAP: fibroblast activation protein
GM-CSF: granulocyte-macrophage colony-stimulating factor
GWAS: genome-wide association study
HCC: hepatocellular carcinoma
HDAC: histone deacetylase
HIF: hypoxia-inducible factor
HMGB1: high-mobility group box 1
IBD: inflammatory bowel disease
ICI: immune-checkpoint inhibitor
IFN: interferon
IL: interleukin
iTAM: iron-rich tumor-associated macrophage
LAM: lipid-associated macrophage
LXR: liver X receptor
M-CSF: macrophage colony-stimulating factor
MGC: multinucleated giant cell
MHC: major histocompatibility complex
MMT: macrophage-to-myofibroblast transition
mTOR: mechanistic target of rapamycin
NASH: non-alcoholic steatohepatitis
NFATC1: nuclear factor of activated T cells 1
NF-κB: nuclear factor-κB
NSCLC: non-small-cell lung cancer
NuRD: nucleosome remodeling and deacetylase
OXPHOS: oxidative phosphorylation
PARP: poly(ADP-ribose) polymerase
PD-1: programmed cell death protein 1
PD-L1: programmed death ligand 1
PDAC: pancreatic ductal adenocarcinoma
PPAR: peroxisome proliferator-activated receptor
RANKL: receptor activator of nuclear factor-κB ligand
SCENIC: single-cell regulatory network inference and clustering
scRNA-seq: single-cell RNA sequencing
SREBP: sterol regulatory element-binding protein
STAT: signal transducer and activator of transcription
TAM: tumor-associated macrophage
TF: transcription factor
TGCT: tenosynovial giant cell tumor
TGF-β: transforming growth factor-β
TLR: Toll-like receptor
Treg: regulatory T cell
TREM2: triggering receptor expressed on myeloid cells 2
UPEC: uropathogenic Escherichia coli
VEGFA: vascular endothelial growth factor A.
